# Toward an Ecological Understanding of Transnational Chinese Language Teachers’ Professional Wellbeing in the United Kingdom

**DOI:** 10.3389/fpsyg.2022.877289

**Published:** 2022-04-26

**Authors:** Isobel Kai-Hui Wang

**Affiliations:** English Language Teaching (ELT) Research and Methodology, Institute of English Studies, University of Graz, Graz, Austria

**Keywords:** Chinese, language teacher, wellbeing, ecology, intercultural adjustment

## Abstract

Given the lack of reports of Chinese language teachers’ wellbeing in the literature, this study aims to investigate the professional wellbeing of eight teachers of Chinese as a foreign language in the United Kingdom based on in-depth, semi-structured qualitative interviews. Interview data provided a rich picture of the rewarding aspects and challenges that they experienced in their professional lives. The main findings revealed the complex interplay of their professional wellbeing and different levels of ecology (i.e., cultural, institutional, classroom, and personal). The study also identified the specific strategies that the teachers deployed for (1) coping with work-related stress and for (2) maintaining wellbeing alongside professional productivity. The paper calls for further research to apply a close-up lens to the wellbeing of foreign language teachers across timescales. The implications for transnational language teachers’ mentoring interventions are discussed.

## Introduction

In recent years, there has been an extraordinary growth in the popularity and importance of Chinese language education on a global scale. In the case of the United Kingdom, since 2000, there have been increasing demands for Chinese language learning from primary education to lifelong learning in the United Kingdom (Zhang and Li, 2020). As key players in Chinese language education, Confucius Institutes (CIs) and Confucius Classrooms (CCs) serve as important platforms providing language teachers to meet the growing demand for learning Chinese in the United Kingdom. CIs are established in collaboration with both Chinese and British partner universities, with a particular focus on tertiary and lifelong education. In addition, CCs are set up mostly for secondary education. The need for qualified teachers of Chinese who are able to perform professionally and effectively in a culturally different context continues to grow (Moloney and Xu, 2018). In the field of Chinese language education, much of the research has been conducted in mainland China to address local needs (Gong et al., 2020). However, comparatively little attention has been dedicated to the professional lives of Chinese language teachers while teaching abroad in a multicultural context.

When teaching internationally, language teachers are likely to suffer additional stressors, such as insecurities about their own language proficiency, dealing with learner diverse cultures, and adaptation to a new culture and institutions (Gregersen et al., 2020). This is especially true of those teachers whose home culture differs significantly from the host culture. Teacher wellbeing has received considerable attention in the mainstream education literature (e.g., Pillay et al., 2005; Parker and Martin, 2009; Aulén et al., 2021), but the psychology of second/foreign language teachers is relatively neglected (Mercer, 2020). Recently, scholars (MacIntyre, 2021; Mercer, 2021) have emphasized the vital role that the professional wellbeing (PWB) of language teachers plays in the quality of teaching and also issued calls for researchers to expand the research agenda.

Research on CIs has tended to focus on their foreign policy and dissemination strategy (Ye and Edwards, 2017), with relatively little attention paid to the professional lives and the intercultural adjustment of their teaching staff. However, studies have shown that teachers of Chinese as a foreign language (TCFL) experience various intercultural difficulties (Jia, 2014; Ye, 2017), as well as emotional challenges, such as anxiety, tension, and depression while working abroad, with evidence from the United Kingdom (Xiang, 2017), the United States (Liu and Sayer, 2016), and Thailand (Lilasetthakul and Ran, 2011). In view of this, it is surprising that there is so little research investigating Chinese language teachers’ wellbeing in the United Kingdom (for exceptions, see Jin et al., 2021a) and we have little understanding of what would help such teachers to flourish in their professional roles. This study aims to address this gap.

## Literature Review

### Professional Wellbeing of Language Teachers

The interpretations of PWB presented in much of the literature (e.g., Klusmann et al., 2008; Fenwick et al., 2018) tend to be limited to its specific dimensions, such as “satisfaction with time off and work-life balance” and “level of emotional exhaustion and job satisfaction.” Increasing importance has been given to individuals’ subjective wellbeing (SWB), since “subjective” factors, such as people’s unique values and attitudes toward happiness and health, appear to play an essential role in individual perceptions of wellbeing (Diener et al., 2017). The construct of SWB consists of personal experiences of positive and negative affect, individuals’ evaluations of their lives, and their judgments about life satisfaction (Diener and Ryan, 2009). Taking this perspective helps us to understand the subjective experience of PWB as to how language teachers feel and think about their professional lives.

While the study of SWB is pertinent for an understanding of PWB, individual subjectivity alone cannot fully address this concept. Building upon Seligman’s (2011) multidimensional PERMA model, Kern et al. (2014) defined PWB in terms of five domains in order to achieve a more comprehensive understanding of the concept, including emotions (i.e., positive and negative emotions), work engagement, co-worker relationships, meaning (i.e., having a direction), and accomplishment. The main finding of their study suggests that in order to achieve a significant level of PWB, employees need to do well across all the five domains.

Drawing on the definitions proposed by the scholars above and recent views on teacher wellbeing (Mercer and Gregersen, 2020), a working definition for PWB of language teachers was established for the purpose of the present study:

Professional wellbeing of language teachers consists of the five main components, comprising (a) positive and negative emotions experienced by language teachers in the workplace and beyond, (b) teachers’ engagement with their professional lives and roles, (c) relationships they build with other people both inside and outside their professional contexts (e.g., teacher-student relationships and family relationships), (d) the meaning and purpose of work, and (e) their accomplishment in language teaching or achievements of their professional goals.

While empirical investigations into the PWB of second/foreign language teachers are still limited, several scholars (e.g., MacIntyre et al., 2019; Mercer, 2020) have identified a series of unique psychological demands and stressors which these teachers tend to undergo, especially foreign language anxiety, weak job stability and security, and high intercultural demands. The view embraced in this article is that we should acknowledge the importance of a subjective personal sense of PWB, but also consider the interrelationship between their PWB and contextual conditions.

### Toward a Holistic Understanding of Teacher Wellbeing

There has been considerable research that focuses on negative aspects of teacher wellbeing, especially on stress and burnout in teachers (e.g., Coulter and Abney, 2009; Wieczorek, 2016; Bottiani et al., 2019). The “negative” side of teacher wellbeing alone, however, does not provide a complete understanding. In order to broaden the current scope of research, there has been increased attention paid to a positive psychology perspective, which includes a consideration of the important role that positive aspects of wellbeing play in helping teachers flourish in their profession (Budzińska and Majchrzak, 2021). With regard to language teachers, in the light of the PERMA model (Seligman, 2011), factors have been identified to specifically contribute to their wellbeing, such as greater emotional control and competencies and a positive sense of professional identity (MacIntyre et al., 2019; Jin et al., 2021a). However, Seligman’s model still focuses largely onto wellbeing coming from personal subjective experiences, with no explicit attention placed on the contextual determinants of wellbeing (Mercer, 2021).

To explore the complexity and situatedness of teacher wellbeing further, scholars (e.g., Cross and Hong, 2012; Mercer, 2020) have been particularly attracted to the ecological examination of wellbeing, since this perspective appears to help understand how teacher wellbeing is shaped by a complex array of factors at different systemic levels, such as personal, institutional, and sociocultural levels. Based on the Bronfenbrenner’s (1979) ecological system theory, an ecological perspective involves investigating teachers’ wellbeing not merely within the individual, but also within the broader ecological system, including (a) the microsystem (e.g., the ecology of the class), (b) the mesosystem referring to interrelations between different contexts (e.g., the relationship between the ecology of the institution and the ecology of personal life), (c) the macrosystem referring to the broader social, cultural, and political setting (e.g., the cultural ecology), and (d) the chronosystem reflecting the effects of time (e.g., teacher career span). This ecological perspective contributes to an in-depth understanding of the distinctive characteristics of the subject-specific domain (Jin et al., 2021b). Regarding language education, Hofstadler et al. (2020) focused on the context of Content and Language Integrated Learning (CLIL) and identified critical personal and contextual factors that mutually affected the PWB of CLIL teachers, such as national and institutional policy, workload and teaching materials, and personal benefit from teaching CLIL. A further, related insight into the PWB of Chinese language teachers at secondary-school level in the United Kingdom was provided by a qualitative study by Jin et al. (2021a), in which the researchers found that their PWB was situated within four particular ecologies: the ecology of the school, the ecology of work and life, the ecology of the education system, and the societal ecology of modern foreign languages. Based on an eco-systemic analysis, tensions between teacher work and life, lack of family support, and job insecurity were identified as the crucial factors threatening Chinese language teachers’ PWB.

It is clear that both ecological and positive psychological perspectives have value which contributes to a holistic understanding of Chinese language teachers’ PWB. The current study was informed by an ecological perspective which enabled the exploration of not only the individual nature of Chinese language teachers’ PWB, but also its contextually situated characteristics across different levels of their ecology. A positive psychological perspective also helped understand the sources of their positive wellbeing without neglecting the challenges teachers encounter in their professional lives.

### The Intercultural Experiences of Confucius Institute Teachers

Previous studies have shown that transnational teachers tend to encounter considerable challenges, such as the language barrier, culture shock, and relationships with host national colleagues (Roskell, 2013; Ding, 2018). In common with transnational teachers in general, CI teachers also appear to face the similar difficulties associated with their intercultural adjustment, giving rise to their psychological stress (Jia, 2014). Scholars have shown particular interest in CI teachers’ distinct professional experiences and highlighted a number of key challenges specifically experienced by this group of teachers, including weak psychological and communicative competences, a lack of suitable teaching materials, limited professional development opportunities (Shei, 2020), and inequality in the distribution of workload (Bao et al., 2020).

**FIGURE 1:**
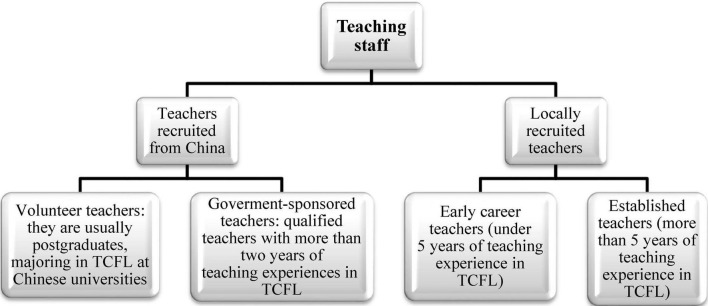
The composition of the teaching staff at Confucius Institutes.

In most CIs, the teaching team consists of three types of teachers: volunteer, government-sponsored, and locally recruited teachers. These teachers have very diverse educational backgrounds and vary greatly in their teaching experiences (see Figure 1).

Volunteer teachers (VTs) who are recruited from China are an important source of the teacher supply for CIs. The number of VTs was found to be significant, with a total of 47,000 by the end of 2018 (Wang and Zhang, 2021), and thus a number of studies have focused on their challenges while working abroad. For example, Zhang (2018) showed that VTs suffered from a wide range of stressors, such as lack of teaching experience, low professional status, lack of professional recognition, and shifting identities. Their sense of self-efficacy, especially their beliefs about their ability to manage a class and communicate with local students, played an important role in influencing their autonomy. In another study, Liu and Dervin (2016) also found that VTs tended to have lower self-efficacy, compared with government-sponsored and locally recruited teachers, which in turn affected their interpersonal behaviors toward their students. They suggested that VTs’ self-efficacy beliefs should be understood in relation to a wider social context in terms of the degree of acceptance by the host society and their social economical status. These contextual factors were also related to their job satisfaction and perceptions of intercultural experiences.

Limited competence in the host language has been identified as a major obstacle to CI teachers’ effective communication both inside and outside the classroom (Chen and Springer, 2020). In addition, particularly for VTs and government-sponsored teachers (GTs), evidence suggests that many of them have limited training in localizing Chinese language pedagogy, leading them to feel challenged, sometimes even frustrated, in their efforts to cope with incongruence between their teaching beliefs and actual classroom practices (Wright, 2020). Li and Tucker (2013) also pointed out that some locally recruited teachers (LTs) were not equipped with sufficient subject knowledge in Chinese linguistics.

Although CI teachers face a variety of issues while teaching abroad, scholars (e.g., Ye and Edwards, 2017) have stressed the benefits of teaching Chinese in transnational contexts, including the development of pedagogical and intercultural competences. A range of factors have been identified as contributing to their professional growth and wellbeing, such as continuing professional development opportunities (Zhang and Li, 2020), professional resources (Bao et al., 2020), institutional support (Ye, 2017), and the cooperation of key stakeholder, i.e., peer colleagues, directors from both China and foreign partner institutions, and teacher educators (Liu and Dervin, 2016).

### Rationale for This Study

The takeaway from the review of literature is that the evidence is not yet available to understand what critical personal and contextual factors are contributing to or detracting from the PWB of transnational Chinese language teachers in multicultural contexts. The aim of this study is to add to this body of research and enrich our understanding of their professional lives by taking an ecological perspective. The focus of the current study was on CI teachers who teach Mandarin through the medium of English in an intercultural context. Teacher training programs offered for CI teachers tend to focus on Chinese teaching pedagogies, cultural and educational knowledge of the host country, and Chinese cultures (Shei, 2020), but those teachers are seldom provided with explicit training in how to develop their wellbeing competences and cope with their work-related stress. There is a critical need to provide them with guidance as to how to promote their PWB alongside their professional productivity.

## Research Questions

The following research questions were posed to guide this study:

1. How do Confucius Institute teachers in the United Kingdom experience their professional lives?
  a) Which aspects of their professional lives do they find particularly rewarding?
  b) What challenges do they encounter in their professional lives?
2. What factors do they perceive affecting their professional wellbeing from an ecological perspective?
3. In what ways do they manage their professional wellbeing?

## Materials and Methods

A qualitative approach was chosen to explore CI teachers’ PWB. Since wellbeing implies individuals’ perception and subjective evaluation of their lives, the value of qualitative inquiry has been increasingly recognized in capturing PWB’s unique characteristics and personal subjectivities (Clark, 2012; Mercer, 2020). This study employs narrative inquiry (Clandinin and Connelly, 2004) as a methodology, which is a way of understanding teachers’ lived experiences through the study of *interaction* (personal and social), *temporality* (past, present, and future), and *situation* (place). In the case of the current study, a narrative pathway was followed to reflect the *temporality* of experience. The researcher started by inquiring about participants’ past teaching experiences and then moved to their current experiences, as well as their future plans. The dimension of *situation* was considered in connection to the dimension of *interaction*. The researcher looked beyond the individual and sought feedback from participants on how their wellbeing was related to ecological conditions (e.g., their students, class, workplace, and the host culture). Narrative inquiry chosen for this study is consistent with an ecological approach, which encouraged the investigation of language teachers’ PWB from a situated, temporal perspective–dynamic interactions among teachers’ PWB, their social and personal lives.

### Context and Participants

In order to recruit participants, the researcher first approached some CIs across the United Kingdom. One of the CIs agreed to forward a recruitment letter to their teachers. This CI was located in a British university. It is one of the earliest and biggest CIs launched in the United Kingdom, comprising eight members of teaching staff, including five volunteer teachers (VTs), two government-sponsored teachers (GTs), and one locally recruited teacher (LT). All the CI teachers were Chinese nationals and indicated their willingness to participate in this study. In order to capture a diverse sample, all eight teachers were selected through criterion sampling, based on three criteria, including different types of teachers (i.e., VT, GT, and LT), career stages (i.e., beginning teachers who have less than 1-year teaching experience, early career teachers who have less than 5-year teaching experience, and experienced teachers who have over 5-year teaching experience), and willingness to participate in the project.

The VTs were all post-graduate students, majoring in TCFL at the same Chinese university. Both GTs had prior experience of teaching Mandarin at CIs outside China. The LT (Lan) had 8 years of teaching experience at two British CIs. At the time of the study, most of the teachers taught adult learners at the CI, and only one GT (Mei) worked for CCs at the secondary level in the United Kingdom. The seven teachers who were directly recruited by Hanban (the Confucius Institute headquarters in Beijing, China) obtained a teaching certificate in TCFL and the locally recruited teacher held both a TCFL certificate and the Post-graduate Certificate in Education (PGCE, i.e., a required qualification to teach in state-maintained schools in England). An anonymized overview of the participants’ biographical information is provided in Table 1.

**TABLE 1:** Participants’ biodata.

Pseudonym	Sex	Age	Type of teachers	Chinese teaching experience	Previous time spent in foreign countries	Teaching qualification
Jia	F	23	VT	7 Months	0	Cert TCFL
Yue	F	23	VT	8 Months	0	Cert TCFL
Fu	M	24	VT	1 Year	0	Cert TCFL
Fei	F	25	VT	7 Months	0	Cert TCFL
Yi	F	26	VT	1 Year	0	Cert TCFL
Wei	M	27	GT	4 Years	2 Years	Cert TCFL
Mei	F	33	GT	10 Years	5 Years	Cert TCFL
Lan	F	38	LT	9 Years	8 Years	Cert TCFL and PGCE

### Instrumentation and Data Collection

In this study, data were generated from a biodata questionnaire, informal interviews, and in-depth, semi-structured qualitative interviews with eight CI teachers of Mandarin Chinese. Information on individual teachers’ backgrounds, career trajectory, as well as the CI’s current practices was first collected from a biodata questionnaire and an informal follow-up interview. Next, the semi-structured interviews were conducted to explore the following major themes:

• intercultural adjustment;
• pedagogical adaptation;
• overall wellbeing;
• working climate/environment;
• work/life balance;
• professional identity;
• professional and personal goals.

An interview protocol was designed with three main sections, including participants’ past experiences (i.e., career trajectory), their present professional lives in the United Kingdom, and future selves. It was constructed with its questions and probes based on a review of the literature dealing with an ecological understanding of teacher wellbeing (Cross and Hong, 2012), positive aspects of teacher wellbeing (Gregersen and MacIntyre, 2018) as well as expatriates’ intercultural adaptation (Zhu, 2014).

The interviews were conducted individually with each participant in their mother tongue, Chinese. Each semi-structured interview session lasted approximately 60 min in length. All interviews were transcribed by the researcher and pseudonyms were assigned to each participant. 10.33 h of interview data were transcribed and a corpus of 92,850 words was generated in total. The researcher translated the transcriptions into English and checked back with the participants regarding any unclear points emerging from the translation.

### Data Analysis

In order to ensure that the interpretation remained faithful to the participants’ original ideas, the interview data were analyzed based on the participants’ actual language using the qualitative data analysis software ATLAS.ti. Multiple waves of coding were operated to ensure the detailed examination of the participants’ lived professional experiences (Smith, 2017). The first stage involved close reading of each interview transcript several times to gain a thorough understanding of the data. Next, a direct and explicit analysis of data was performed and this process involved a line-by-line coding which allowed an intensive engagement with the data as well as unanticipated elements to emerge, as suggested by Charmaz (2015). The third wave of coding was used to organize the initial codes in order to look for connections and themes and then to generate a preliminary list of categories. The fourth wave of coding was refined in collaboration with a specialist in teacher psychology, and this was used to reach agreement on coding and develop categories with a view to enhancing the interpretation of various ecological factors affecting the participants’ PWB and their reported strategies for managing PWB.

## Results

The first section provides an overview of the positive aspects and challenges that the Chinese language teachers experienced in their professional lives before turning to an in-depth analysis of factors contributing to their wellbeing.

### Experiences of Chinese Language Teachers in Their Professional Contexts

#### Rewarding Experiences

**FIGURE 2:**
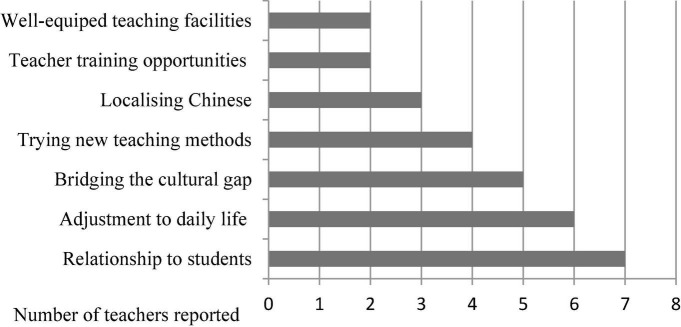
The sources of positive affect.

**FIGURE 3:**
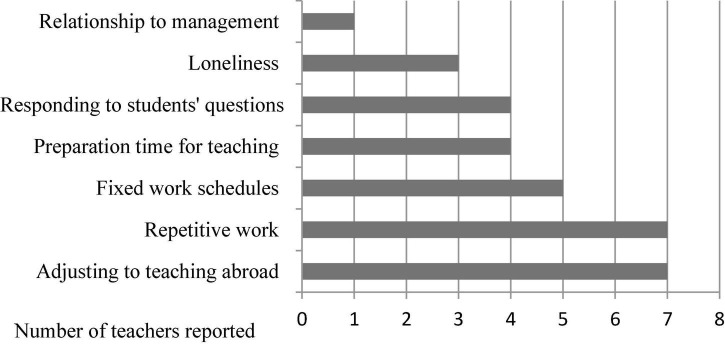
The sources of negative affect.

The narratives of the eight teachers showed the sources of enjoyment and satisfaction as well as the sources of stress and dissatisfaction the teachers reported in their professional lives (see Figures 2, 3).

With regard to the sources of positivity, most of the teachers stressed that their harmonious, healthy relationships with their students were the most rewarding aspect of their professional lives while working in the United Kingdom. In particular, their stories involved great pleasure and satisfaction derived from gaining the respect of their students:

Sometimes my English is not good enough to address their questions clearly, but they never embarrass me. Instead, they really listen to my explanation, and help me formulate my ideas in English. (Yi/VT)

In describing the joy from their teaching, the teachers highlighted a harmonious relationship to their students, since they explained that teachers tended to be highly respected in Chinese society and students rarely confronted their teachers in China. The respect and support they gained from the local students, especially from senior adult learners, also played an important role in helping them develop into more confident teachers during their initial contact with the host culture, and positively contributed to their PWB.

When asked to describe the experiences they enjoyed in the teaching profession, the two experienced teachers, Lan/LT and Mei/GT, pointed out that they derived positivity specifically through establishing an inspirational, enduring relationship with their students. For example:

I enjoy learning by teaching. 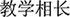 [We learn from each other]. Not only do I teach my students and see them grow, but also I can learn from their perspectives and culture. (Mei/GT)

Mei acknowledged the value of this collaborative relationship between students and teachers and considered it as an important element contributing to her motivation and commitment to teaching.

In their narratives, another outstanding positive experience highlighted by the teachers was related to teaching Mandarin alongside the Chinese culture, especially topics and elements relevant to present-day China. Several teachers reported that they achieved better mutual understanding and cross-cultural acceptance through sharing authentic Chinese culture, as well as learning the host culture from their students. Fu’s account of enjoyable experiences at work provided an example:

Both Chinese teachers and local students might hold cultural assumptions and stereotypes before starting the course. I believe that interaction is a two-way process. I found joy in seeing us value both cultures. (Fu/VT)

Later in their teaching career, the two experienced teachers, Mei/GT and Lan/LT, also described their positive experiences of localizing content and pedagogies for local students, for instance, Lan expressed:

Every CI should have its unique feature, since they locate in different regions and sociolinguistic contexts. I feel very proud of our CI, because we are trying to create our own materials to meet local students’ needs. (Lan/LT)

Two of the VTs also recognized the educational value of using the localized materials to maintain students’ motivation to learn Chinese, and expressed pride in the materials that they creatively developed.

Their narratives revealed that their professional and personal lives did not exist in isolation. The teachers were satisfied with various aspects of daily life in the United Kingdom (e.g., a large Chinese community, fitness facilities, and public parks). Most of the teachers emphasized that their early adjustment to their daily life was relatively smooth and this also eased their adjustment to their new role at the CI, contributing to their job satisfaction.

#### Challenges

With regard to the sources of negativity (see Figure 3 above), apart from the LT, the anecdotes of the seven teachers suggested a range of stressors associated with adjusting to their new teaching context during their initial stage of the sojourn, such as teacher-student interaction, local students’ learning styles, and the class size. In particular, when the teachers were asked to describe the key challenges of working in the United Kingdom, four of them reflected on considerable cultural differences in classroom interactions they experienced while teaching. For example, Fu/VT commented:

The British students tended to initiate communication and ask critical questions […] We tended to ask students to remember certain grammatical rules in China, but here they liked to ask why.

Fu also stated that Chinese students tended to follow and obey their teachers’ instruction, for example, memorizing grammatical rules without doubt. By contrast, he found that British students were more likely to challenge teachers’ pedagogical decisions and express their doubts to him. As a consequence, he had to respond to some “unexpected” questions in every lesson and found those situations particularly stressful. Like Fu, several VTs also shared similar experiences and indicated that they tended to put pressure on themselves before responding to students’ questions and considered that their face might be threatened if they failed to address them well. They described not only their negative experiences during teaching, but also the source of stress beyond the classroom. For example, most of the teachers experienced negativity due to the large amount of time spent on repetitive tasks. They stressed that adding annotation with learning materials in Pinyin [Romanized spelling for Chinese] on a daily basis resulted in not only boredom at work, but also a lack of time to work on other duties. By contrast, through her stories, the greatest source of stress for the locally recruited teacher, Lan, was handling with the relationship to the Chinese director. She described her situation as follows:

Firstly, I found it difficult to get close to the Chinese director. Secondly, differences in our teaching approaches and styles created so many conflicts between us.

In addition, the five VTs’ account of negative experiences at work also showed that a major source of job dissatisfaction was derived from their fixed work schedule. Prior to working full time at the CI, they were post-graduate students in China and had more freedom to plan their own time. The transition from university to their professional life was challenging. For example, Jia stated, “I’ve felt demotivated and less productive when I’m tied to my desk from Monday to Friday.” In addition to the fixed hours on weekdays, those who taught on Saturdays experienced more conflicts between work and private life. The four VTs who had very limited teaching experience tended to spend considerable time designing their lessons. They also suffered from work–life imbalance problems.

Four of the CI teachers also commented that they suffered from great loneliness due to a lack of supportive friendships in the United Kingdom. This resulted in their low level of satisfaction with working abroad. Consequently, they stated that they might continue to teach Chinese in China but would not work abroad in the future.

### Factors Affecting Professional Wellbeing

#### Language-Related Issues

With the exception of the locally recruited teacher, the seven CI teachers highlighted in their narratives that they all suffered psychological strains while working in the United Kingdom, such as stress and anxiety, from teaching Chinese through the medium of English, as well as communicating with local people in English. For example:

I got nervous when my students asked me questions. I was really worried that I couldn’t explain my answer clearly in English. (Fei/VT)

I felt very stressful to attend weekly meetings with my British colleagues, because participating in their discussion and understanding their humor were very challenging. (Mei/GT)

##### Institutional and Personal Ecologies

Prior to working at the CI, most of the teachers, except for the LT and the GT who worked for the CCs, had little or no direct contact with native speakers of English. They explained in the interviews that their core team members at the CI were all Chinese nationals and had little opportunity to interact with British colleagues. They also expressed that their social network was monocultural beyond their workplace, and thus their speaking skills in English were less likely to develop from socialization with members of the target language community. Jia/VT articulated:

Before I came to the United Kingdom, I had expected to meet more local people and have more opportunities to speak English in the workspace and beyond. Yet, I now realize that apart from teaching I still spend most time speaking Chinese, since my colleagues and flatmates are all from China. Personally, I’m not outgoing and feel anxious to make friends with British people.

##### Class and Personal Ecologies

Although some VTs felt nervous when talking to local students during their initial period of sojourn, as previously noted, the support they gained from their students enabled them to become more confident in their use of spoken English. Three VTs also described instances in which they invested a high level of personal effort to develop their English language skills during their early adjustment. However, throughout the data, Jia and Fu also stated that they were not satisfied with their progress in English over time, which had been less than expected, and this mismatched expectation negatively affected their wellbeing in general. For example, Fu/VT began by explaining his expectation before coming to the United Kingdom and then described how disappointed he felt with his limited English language skills after working one year in the United Kingdom:

I expected to speak English fluently when I return to China. This was one of the main reasons why I chose to work in the United Kingdom. Every time when I think about how little progress I have made in my English, I feel very frustrated and disappointed.

#### Intercultural Adjustment

##### Cultural Ecology

The interview data indicated that adjustment to changes in their new work, social, and daily lives was a critical factor that influenced the teachers’ professional wellbeing. Apart from the locally recruited teacher, other CI teachers all reported that they suffered from a range of stressors while teaching and living abroad, such as:

• adapting their pedagogies to make their teaching useful for local students,
• experiencing differences in British and Chinese conceptions of the teacher-student role relationship,
• and establishing new social contacts in the target community.

The transition from student teacher to beginning teacher and coping with an independent life in the United Kingdom were additional stressors for the VTs during their early adjustment. For example, in their narratives, Fu and Yi described their experiences as follows:

My first 3 months in teaching was undoubtedly stressful. I found that there was a big gap between what I had learned about the principles of teaching Chinese and the realities of teaching. In the real classroom, you have to cope with all sorts of issues by yourself. (Fu/VT)

It was my first time to live independently in a different country. During the first couple of weeks, I had been incredibly busy and stressful. (Yi/VT)

However, their intercultural experiences also enabled them to develop professionally and personally over time. For instance, through her stories, Yi recounted instances in which interacting with local students helped her look beyond cultural stereotypes, and grow in terms of skills in teaching foreign students, self-efficacy in the English language, and intercultural sensitivity. The perceived professional development and personal growth were inextricably linked with their PWB and job satisfaction. As Fu/VT noted:

Working in a different country with a very different culture from mine has become a rewarding experience for me. I have gained great pleasure and satisfaction from my professional development.

##### Institutional Ecology

Six teachers found both their pre-departure training program and initial on-arrival orientation workshops helpful for their work and general adjustment in the United Kingdom, regarding practical information on social etiquettes in the host culture and the methodology of TCFL. However, five of them also stressed that there were still limited professional development opportunities available at the CI, especially as to intercultural competences and localization strategies for teaching Chinese. When the researcher asked participants to describe their professional goals for the future, Jia/VT described her situation in the following terms:

I’m eager to get some further training provided by more experienced local teachers while working here, in terms of teacher strategies for motivating local students to learn Chinese characters and strategies for adapting Chinese language teaching materials.

Echoing Jia’s position, other VTs also stressed that they expected to receive ongoing on-site training and considered to be important for their effective functioning in the classroom, contributing to their positive emotions and job satisfaction. Even though three teachers mentioned that they were allowed to attend training sessions across the United Kingdom, rigid working schedules limited their opportunities to attend these off-site training sessions.

One the one hand, in their narratives, the eight teachers shared their concerns and challenges in their professional lives. On the other hand, they also pointed to the fact that institutional and peer support played an important role in facilitating their adjustment to daily living. All the VTs highlighted the supervisory role that the Chinese director played, and appreciated her help to ease their transition from being a student to being a teacher. Yet, two of the VTs further commented that the suggestions provided by the Chinese director on teaching approaches, e.g., “a combination of the grammar translation and audio-lingual” were inconsistent with those provided by the locally recruited teacher, e.g., “the communicative approach.” This created uncertainties and confusion as to which approaches would be more appropriate to teach local students.

##### Personal Ecology

The teachers reflected on their cross-cultural experiences and highlighted a number of important individual variables related to their intercultural adjustment, especially their previous experience abroad, knowledge about the target culture, spirit of adventure, and openness toward differences. As Yue/VT noted:

Although I had never been to other countries before, I was able to take on unfamiliar challenges and open to new ideas. I tried to talk to strangers to improve my English and also enjoyed knowing their cultures. I think all these personal qualities were very helpful during my early adjustment in the United Kingdom.

Their personal characteristics, such as extraversion, conscientiousness, and self-direction also led to individual differences in their perceptions of stress while working abroad, and affected subsequent PWB. For example, one teacher explained:

I’m a very outgoing person. I admit that it was stressful to understand local teachers’ English, but I wasn’t afraid to talk to them and express my opinions. (Mei/GT)

#### Teacher Status

##### Cultural Ecology

Chinese has been one of the most popular languages in the United Kingdom. Given the rise of Chinese in British society, most of the teachers (6/8) commented that they received high prestige and enjoyed the respect they gained from their students and local communities during their sojourn. Interestingly, two of the teachers (Jia/VT and Fu/VT) also drew a comparison between their status and the status of international Chinese students in the United Kingdom. They believed that their status was much higher than international Chinese students because they were able to receive financial and welfare benefits from both the United Kingdom and Chinese institutions. Their perceived status in the United Kingdom was considered as another important factor contributing to their intercultural adjustment and job satisfaction.

##### Institutional Ecology

Lan/LT was the only CI teacher who compared the status of CI teachers with those teaching Chinese language courses or degree programs offered by United Kingdom universities:

The teachers working there [at a British university] have a higher status than us. Some students also think that they are more professional. I also know that British universities provide them with longer annual leave days, more flexible hours of work, and a wide range of CPD opportunities.

She highlighted the importance of professional status for her wellbeing, and explained how the lack of job security, professional recognition, and continuing professional development (CPD) opportunities detracted from her PWB. Thus, in order to gain a higher professional status, she considered seeking a lecturer position at British universities at some point in her career.

#### Teacher Self-Efficacy

##### Cultural Ecology

Most of the teachers also revealed that their cultural background affected their personal perceptions of teaching effectiveness. The three VTs (Jia/VT, Yue/VT, and Fei/VT) reflected on their own learning experiences in China and explained that the teacher in China was traditionally viewed as the authority figure and the source of all knowledge. They considered that their “mian-zi” (face) would be threatened or they might lose their respect from their students if they did not know the answers to their questions or were not able to answer their questions correctly. By contrast, the narratives of Wei/GT, Mei/GT, and Lan/LT showed that their experience of teaching Chinese abroad contributed to their higher levels of teaching self-efficacy. In particular, Lan/LT and Mei/GT stressed that considerable experience of teaching Chinese in the local context boosted their self-efficacy and positively contributed their regulation of emotions in the classroom. Lan/LT expressed:

As I gained more teaching experience, I learned various ways to make my lessons more effective and engaging. I could see that students really enjoyed my lessons. This was why I enjoyed teaching so much. I think my job was really meaningful.

##### Class Ecology

Across interviews, the teaching self-efficacy of the CI teachers appeared to be dynamic across context and time. During the initial period of their sojourn, the two VTs pointed out that their sense of teacher efficacy decreased and they suffered high levels of stress because of their students’ critical feedback on their teaching, and these negatively affected their PWB. Yi/VT provided an example:

I never received negative feedback from my mentors when I was a practicing teacher in China. I was so sad when I got negative feedback from local students and felt so stressful during that time.

Six teachers also reported that there was an improvement in their self-efficacy as they accumulated knowledge of the target culture and gained a better understanding of their students’ needs, interests, and learning styles. For example, Yi/VT continued to explain:

I tried to communicate more frequently with my students after class […] I moved toward a more task-based approach which my students favored. I became more confident when I see more students enjoy my class.

Three of the teachers also stated that a growing sense of self-efficacy triggered their positive in-class emotions leading to better teaching performance, especially in terms of flexibility in teaching. Consequently, this also increased their job satisfaction and positive sense of professional identity. As Fei/VT put it:

When I continued to receive positive feedback from my students and colleagues, I felt more confident to teach. I love the job and believe that I can be a good teacher.

##### Personal Ecology

In their narratives, the eight CI teachers described different self-perceptions of their teaching competences in the classroom regarding:

• teaching Chinese in Western settings,
• engaging and motivating learners of Chinese,
• knowledge of the Chinese language,
• and decision-making abilities.

The teachers stressed that confidence and sense of efficacy in their ability to teach Chinese was another contributory factor to their PWB. Compared with the three more experienced teachers, the five early career teachers considered that they had lower levels of self-efficacy, especially during their initial period of sojourn, and their teaching self-efficacy influenced their actual ability to cope with challenges in the classroom, and consequently their PWB. For example, Jia/VT explained:

I tried to plan fewer interactive activities, because I wasn’t confident enough to handle the problems that arose from our real-time interactions. I could get into a panic when a student asked me a challenging question.

Like Jia/VT, most of the VTs also described that they experienced anxiety when they were uncertain how to make appropriate pedagogical decisions. By contrast, the experienced teachers, Mei/GT and Lan/LT stated that they were skillful at dealing with challenging circumstances pedagogically and emotionally, and had a stronger sense of purpose and agency to maintain quality teaching.

The two VTs (Yu and Fu) further commented that although they experienced stress due to low teaching self-efficacy during their early adjustment, such stressful experience also motivated them to improve their teaching skills and positively promoted their adaptation and wellbeing. For example, Fu/VT explained:

Stress was not always bad. I didn’t want to disappoint my students. I spent considerable time preparing my teaching. I also talked to experienced teachers as many as possible and learned useful ways of dealing with unexpected situations.

#### Work-Life Balance

##### Institutional and Personal Ecologies

Five teachers recounted instances in which they were struggling to balance their work and personal life. In particular, their inflexible scheduling and weekend work were important causes of tensions between their personal and professional commitments. Due to their low teaching efficacy, some VTs further stated that they had to spend considerable time preparing for their lessons.

All the teachers highlighted that the Chinese director provided them with practical advice on how to stay active and maintain a work-life balance. Six of the teachers found it helpful for reducing their stress and boosting their positivity, especially through joining the local gym and participating in fitness sessions.

Keeping a fitness routine makes a really difference on productivity and enjoyment at work, and also enhances my health and overall wellbeing. (Yi/VT)

Two of the teachers also reported that they made a further effort to improve their work-life balance, for example by “taking at least one day off from work each week” (Yue/VT), or “finding time to learn new skills, e.g., playing guitar” (Mei/GT), since they found that a healthy work-life balance played an important role in promoting their work happiness and sustained engagement in the profession.

#### Relationships With Colleagues and Confucius Institute Management

##### Institutional Ecology

With regard to the VTs, they all referred to working relationships with their co-national colleagues that were significantly related to their PWB. They described how the CI provided them with a work space in an open-office layout and the senior teaching staff (i.e., Lan/LT) also shared the same office with them. Most of the VTs were very satisfied with this type of office arrangement, as they stated that it fostered a collaborative, supportive working relationship. Lan/LT also noted:

I was there to help the VTs. I encouraged them to ask me questions and raise their issues in an effort to cultivate equal, trusting relationships among us.

Their data suggested that this kind of positive work climate provided the early career teachers with networks of peer support and materials exchange, subsequently contributing to their professional flourishing. However, one teacher found distracted when other colleagues discussed their work in the open workspace.

The two GT teachers (Mei and Wei) shared the same office. In her narrative, Mei described how she spent considerable time working at a local school and teaching CCs, and thus she had little time to interact with the team at the CI. They both commented that they became more socially integrated into their work lives after participating in team projects and social events organized by the CI. Both of them believed that building effective working relationships with their directors and colleagues positively affected their PWB, but they avoided turning working relationships into personal ones.

The Chinese director was a teacher educator and had the opportunity to teach the five VTs and two GTs when they were student-teachers in China. She continued to play a major role in mentoring these teachers while they were teaching in the United Kingdom. Throughout the data, most of the teachers were satisfied with the mentor-mentee relationship; in particular, they stated that the support and advice she provided strengthened their sense of self-efficacy and professional identity. For example, Jia/VT commented:

She [the Chinese director] was not only our boss, but also our teacher. She took care of us. She helped me change from a learner perspective to a teacher perspective.

##### Personal Ecology

One of the VTs, Fu, also mentioned that he was reluctant to seek emotional support from the Chinese director, and he further explained:

Now she [the Chinese director] was my superior. I should not bother her with my personal problems. Instead, I felt more comfortable to seek support from my colleagues.

By contrast, Lan/LT reported that a positive relationship established with the British director enabled her professional growth and commitment to the profession. However, as previously noted, the difficult relationship with the Chinese director, which arose from substantial differences in their educational backgrounds, teaching approaches, and working styles, caused her considerable stress. She also stated in the interview that she received very limited support from the CI as to how to improve their relationship. She felt helpless and expressed her intention of resigning.

### Strategies for Managing Wellbeing

This section reports the most salient strategies that the teachers employed for managing their wellbeing. The strategies are organized according to their function (i.e., coping and thriving).

#### Strategies for Coping With Work-Related Stress

In their narratives, the five early career teachers perceived social support as one of the most useful strategies to help them deal with stressful issues, both professional and personal. They expressed the view that asking their co-national colleagues and the Chinese director for help played an important role in handling stressful changes associated with cross-cultural adjustment as well as their transition from university to work.

During their sojourn in the United Kingdom, three early career teachers also revealed that they preferred to mitigate their personal and work-related stress by seeking emotional support from their family. By contrast, the locally recruited teacher (Lan) stated in the interview that she preferred to seek professional advice from the staff support services offered by the British university. In particular, she was satisfied with the advice obtained on how to manage her relationship with the Chinese director. However, apart from Lan/LT, the interview data showed that the other teachers rarely used the university services, and they explained that they were not clear what specific support services were available on campus and how to access these services.

**FIGURE 4:**
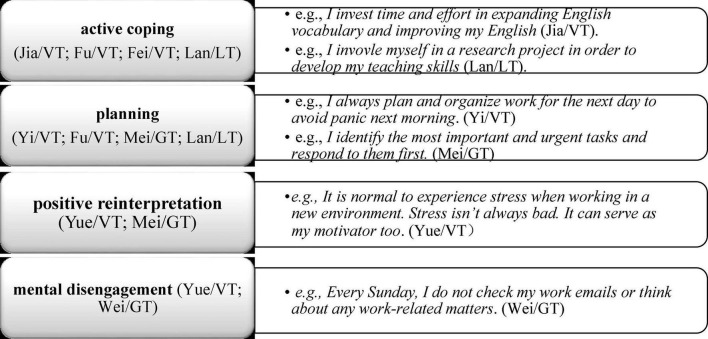
Personal strategies for coping with stress.

In addition to seeking social support, the eight teachers explained that they drew on their psychological resources (e.g., self-regulation) and behavioral resources (e.g., proactive coping) and deployed a range of personal strategies that helped them cope with stress successfully (see Figure 4).

#### Strategies for Promoting Wellbeing

It is clear from participant responses that establishing a work-life balance was one of the major factors promoting the teachers’ PWB. Seven teachers described a number of strategies that they actively employed for achieving a better work-life balance, including:

• integrating physical activities into daily routines,
• choosing and organizing times for relaxation,
• and using mindfulness practice for deep relaxation.

In addition, most of the teachers (6/8) explicitly stated that they deployed strategies for building rapport with students since they believed that positive teacher-student relationships greatly contributed to their PWB, self-efficacy, and job satisfaction. For example, they reported developing their relationships with their students by:

• spending time connecting with students after class (Fu/VT),
• asking students to keep reflective journals which helped her know more about their progress (Lan/LT),
• seeking student feedback on teaching (Yi/VT).

In order to mitigate their in-class psychological strains, three of the early career teachers also explained that they anticipated potential questions that students might ask and prepared for possible responses.

Apart from building rapport with students, another three teachers highlighted the value of using self-reflection strategies with active planning to promote their PWB in their stories. For example, as Yue/VT reflected, “keeping ongoing records of the joys that I experienced at work could activate my positive feelings, especially a feeling of gratitude, to sustain my professional and general wellbeing.” By contrast, Mei/GT stated, “I reflected not only on my positive experiences but also the occurrences that challenged me,” and she further commented that reflecting on past experiences, either positive or negative, helped her adjust goals and plan further action to grow both personally and professionally. Furthermore, Jia/VT and Fu/VT reported the use of downward comparisons as a strategy to generate their positive feelings and emotions. As mentioned above, they tried to notice and appreciate positive things by comparing their experiences with other international students’ intercultural experiences. For example, as Jia/VT put it:

Working abroad is not easy, but we are luckier than overseas Chinese students because we get full support from Hanban. I feel satisfied and grateful.

## Discussion

### Interpretation

The current study focused on the PWB of eight Chinese language teachers and their intercultural experiences in the United Kingdom. While previous studies on teacher wellbeing tended to focus on the negative dimension of experience, the findings from this study underscored the importance of positivity. Regarding the first research question, the present data showed various sources of positivity that fostered the language teachers feelings of enjoyment and satisfaction about their job. The findings suggested additional uplifts that were likely to contribute to their PWB, such as engagement in an integrated approach to teaching content and culture (see also Hofstadler et al., 2020) and forming a collaborative learning environment (see also Mercer and Gregersen, 2020). Being a CI teacher also generated its specific source of positivity, for example, the status of Chinese language education in the United Kingdom, appreciating the support gained from both Chinese and British institutions, and recognizing the value of working in a transnational context. With regard to negativity, the study identified the CI teachers’ unique sources of stress, including work schedule strains, adjusting cultural differences in classroom interactions, and relationship to management. The findings are interesting in revealing that CI teachers’ perception of the source of stress fluctuated across time, and some factors (e.g., self-efficacy and intercultural encounters) which threatened their PWB also had the potential to strengthen their wellbeing depending on individual teachers’ specific circumstances.

**FIGURE 5:**
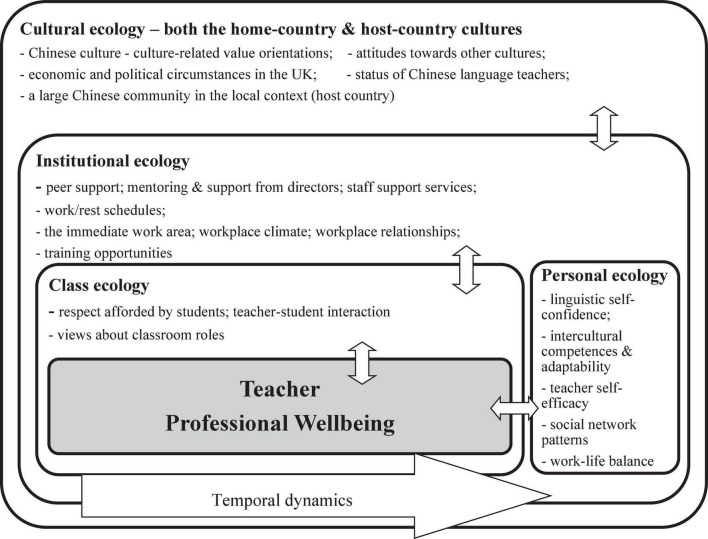
Contributory factors to professional wellbeing.

With regard to the second research question, the findings from this study revealed a plethora of intrapersonal and contextual factors which contributed to the Chinese language teachers’ PWB within and across four different systemic levels (i.e., cultural, institutional, classroom, and personal, for further details, see Figure 5 below). It should be noted that the factors presented below showed those salient in the data and indicated the types of factors that can contribute to teacher wellbeing rather than offering a comprehensive summary of factors. While a number of studies (e.g., De Costa et al., 2020; Gkonou et al., 2020) took a closer look at factors affecting language teachers’ wellbeing at certain levels of their ecology, such as heavy workload, relationship to colleagues and leadership, and their salary at the institutional level, these studies did not explore the interplay of personal and contextual factors affecting wellbeing across different levels. Hence, I would consider this study as a valuable example to provide an ecological understanding of Chinese language teachers’ professional lives and indicate how their PWB as a situated emergent state emerged from the dynamic interaction of personal perceptions and ongoing experiences across different multi-level ecological contexts. This is consistent with the finding from the study by Hofstadler et al. (2020) that examined teachers’ professional lives in their home country and highlighted the importance of class, institutional, and personal ecologies and how they were related to teacher wellbeing. The findings from the current study add a cultural dimension to our understanding of teachers’ PWB. Given that the Chinese language teachers moved from the Chinese to the British context, their intercultural experiences showed a more complex connection with their PWB. Adjusting to the host culture brought various challenges, such as language matters, differences in daily and work routines, and limited knowledge of local pedagogies, which threatened their PWB. Meanwhile, their professional development and personal growth took place through the adaptation process, contributing to their subsequent wellbeing.

In a recent study investigating the wellbeing of Chinese language teachers at secondary-school level in the United Kingdom, Jin et al. (2021a) found that long-term teacher sojourners experienced stress, frustration, and disappointment due to challenges in adjusting to the British education system and cultural norms regarding the teaching profession. In contrast, the current study provided rich insights into the professional lives of short-term teacher sojourners and revealed particular challenges which they encountered during their initial contact with the host culture, such as cultural differences in communication styles and perception of teacher-student roles.

The classroom as a site of intercultural communication is reflected in the data gathered (see also Zhu, 2014). The results of the study suggested that aspects of the class ecology (e.g., teacher-student interaction patterns and classroom roles) and aspects of the cultural ecology (e.g., culture-related value orientations and attitudes toward other cultures) appeared to be closely related. Most of the teachers in this study reported deriving positivity from their harmonious relationships with local students. This could be due to the fact that harmony is considered as the core value of Chinese culture that can influence Chinese people’s attitudes toward preferred communication behaviors and styles of interaction (Wei and Li, 2013). As the teachers increasingly interacted with their students, the data indicated that both parties developed their intercultural sensitivity and tolerance. In particular, mutual respect and understanding seemed to be important for the quality of student-teacher relationships, which remained a critical factor in promoting teachers’ self-efficacy, wellbeing, and job satisfaction (see also Frenzel et al., 2016).

This study provides a useful insight into the ecology of TCFL in multicultural contexts as well as some specific characteristics of teaching Chinese in the CI. In terms of the institutional level, arranging open workspaces, establishing a peer buddy system, and undertaking team projects were identified as important ways to foster connection and collaborative staff relationships. Institutional and peer support at part of the institutional ecology played an important role in facilitating the CI teachers’ adjustment to daily life in the United Kingdom. However, they needed to have further training opportunities on the site to develop their professional skills, as well as more practical advice on enhancing PWB. This finding echoes Chen and Springer’s (2020) observation that many pre-departure training and on-arrival orientation programs did not have a significant impact on TCFL teachers’ professional adaptation due to the fact that they provided little or no training in practical skills which can be used to deal with the specific issues encountered in their overseas teaching practices.

The data also demonstrated the interpersonal dynamics among VTs, GTs, the LT, the Chinese director and the British director. In particular, the findings of this study highlighted the criticality of the relationship to the Chinese director in PWB. The early career teachers (VTs) highly appreciated the supervisory role that the Chinese director played in their work and personal lives, since this fulfilled their expectation of the role relationship in superior-subordinate relations (see also Ye, 2017). By contrast, the working relationship with the Chinese director seemed to be a key source of stress to the LT. Unlike other teachers, the LT, as a long-term sojourner, reported a higher degree of acculturation to the British culture. As a consequence, different working styles, teaching beliefs and approaches tended to cause uneasiness and tension between the LT and the Chinese director. A number of studies have also shown that there were disagreements and tensions between mainland teachers and local teachers, and between Chinese directors and British co-directors, due to cultural differences, and different education backgrounds and teaching experiences (e.g., Xiang, 2017; Li, 2019). Research has highlighted the importance of teacher leaders’ leadership behaviors for teacher wellbeing, work engagement, and job satisfaction (e.g., Cherkowski, 2018). Hanban tended to provide in-service training merely for foreign directors with a particular focus on the Chinese language, Chinese philosophy, and the Chinese culture (Zheng et al., 2019). However, both Chinese directors and foreign directors often receive little or no training in management and leadership skills regarding how to manage intercultural collaboration between Chinese and local colleagues and how to promote teacher wellbeing (Li, 2019).

At the personal level, the results of this study suggested that the lack of English-language proficiency and confidence in teaching Chinese through English appeared to be the critical factors inhibiting not only their adjustment to a new teaching environment, but also their PWB. The teachers’ socio-emotional competences, especially social skills and strategies for managing negative emotions, also appeared to play a crucial role in developing positive relationships and rapport with their students and colleagues (see also Gkonou and Mercer, 2017).

Furthermore, the findings of this study revealed the temporal nature of PWB. The teachers encountered various challenges at work and life and suffered a higher level of psychological strains during their early adjustment, but the experiences of teaching abroad they gained enabled them to grow professionally and personally over time. Therefore, in addition to their individual variation, the interaction of the diverse factors identified in this study (e.g., intercultural adjustment, linguistic self-confidence, self-efficacy in teaching, and work-life balance) across time appeared to contribute toward temporal variations in their PWB.

The data indicated some homogeneity among the eight Chinese language teachers, for example, in terms of the source of positivity and negativity, and strategies for managing their work-life balance. In particular, those who had similar teaching and educational backgrounds shared many commonalities, especially among the five early career teachers. However, this study also showed considerable individual differences in their professional experiences, such as their unique stressors, specific rewarding aspects of their professional lives, preferences as to the work environment, and their perceived functions of their social networks. Therefore, it is important to understand the teachers’ PWB with reference to the interrelationships among their individual difference factors (e.g., personality, agency, and experience of working abroad), their immediate and broader ecologies (e.g., their institutional and peer support, relationship to management, and teacher status in the United Kingdom).

With regard to the third research question, social contact was identified as a major coping strategy for dealing with work-related stress among the eight teachers. A notable body of research has shown the importance of social support for teachers’ psychological wellbeing (e.g., Kinman et al., 2011; Väisänen et al., 2017; Jin et al., 2021a). This study further revealed the function which different sources of social support served. Apart from coping with negative experiences, sustaining a work–life balance and building positive relationships appear to be two key strategies for promoting teacher wellbeing and helping teachers flourish in their roles (see also Mercer and Gregersen, 2020). In this study, the Chinese director played an important role in expanding repertoire of strategies for managing a work–life balance. However, only a few teachers were able to draw on their psychological resources (e.g., self-reflection, gratitude, and positive thinking) to proactively promote their wellbeing. One possible reason would be that very few programs teach teachers wellbeing competences explicitly (Mercer, 2021). Consequently, they might not be aware of their available psychological resources and have little knowledge about strategies for maintaining positive wellbeing.

### Implications for Professional Development

In light of the findings from this study, suggestions are provided for practitioners in the design of future training interventions in order to help CI teachers flourish in their professional roles. Firstly, it would be useful to incorporate social emotional training into traditional pre-departure intervention programs in an effort to help teachers become more socially and emotionally competent in an intercultural context. In particular, it could be beneficial to integrate strategy instruction into training programs, involving raising participants strategic awareness and expanding their strategy repertoire, such as by the development of strategies for quality relationships at workplace and strategies for regulating their emotions.

Besides general orientation programs, this study suggests that teacher sojourners need more adequate pre-sojourn preparation designed to heighten their awareness of cultural differences (e.g., role relationships, linguistic and non-linguistic conventions) and develop strategies for coping with culture shock during their early adjustment (e.g., self-mentoring and minimization of difference). CI program organizers may wish to consider incorporating strategies-based language instruction as well as more intensive English language courses into their pre-departure training programs, especially in terms of strategies for intercultural communication (e.g., active listening and language adjustment) and English for teaching purposes (e.g., responding to students’ questions, organizing small group discussion, and explaining language points).

Another practical suggestion arising from the study concerns the limited CPD opportunities for CI teachers. CIs may need to work collaboratively with local partner universities and provide ongoing in-service training on teacher competences with respect to teaching intercultural groups, localization strategies for teaching Chinese, and strategies for promoting a work–life balance. Opportunities could be also provided for teachers to explore their available resources (i.e., psychological, social, and behavioral) and utilize the resources to cope with their stress. Drawing on a positive psychology perspective, reflection can be a useful way to empower teachers and help them appreciate the positive dimensions of all aspects work-related (Mercer and Gregersen, 2020). Teachers could be encouraged to reflect on their joys, rewarding experiences, and successes that they have had in their teaching, workplace, and daily life during the sojourn as well as the post-sojourn period by means of self-reflection questions or self-accessed CPD materials. In light of the notion of appreciative inquiry (Hammond, 2013), it could be also helpful to involve teachers in teacher research and give them opportunities to identify their strengths in their teaching and find ways of leading to greater success in that area.

Additionally, apart from training programs for teachers, CI program organizers and partner universities may need to provide pre-service training for Chinese and British directors in how to work across cultures, interact with different types of CI teachers, create strong social ties within the team, and develop their understanding of the issues raised by staff.

## Conclusion

This study applied a close-up lens to the intercultural experiences of eight Chinese language teachers in the United Kingdom, with a particular focus on their PWB. A holistic interpretation of the results sheds light on the complexity of their PWB and reveals dynamic interactions between their personal factors and a nexus of contextual factors. The cases demonstrated the important impact of PWB on their commitment to work, job satisfaction, and general wellbeing while also providing insights to inform more effective training and mentoring interventions. Although this study focused on teachers of Mandarin Chinese, the rich nature of the qualitative data provides important insights into teachers’ PWB that can extend beyond to teachers of other languages and facilitate teacher mobility across countries. Given that issues of teacher wellbeing are under-researched in the language education field in general, the findings suggest that more in-depth investigations into the diverse teacher perspectives on their experiences of wellbeing could provide valuable insights into how foreign language teachers at different stages of their careers can be supported with the aim of promoting and sustaining their PWB. Future research would also benefit from an ethnographic case study approach, involving onsite observations, photo-elicitations of lived professional experiences, and video- and audio- recorded interviews during teacher sojourners’ adjustment process, to trace their actual changes and capture the dynamism of teacher wellbeing over time.

## Data Availability Statement

Datasets are available on request: the raw data supporting the conclusions of this article will be made available by the authors, without undue reservation.

## Ethics Statement

The study involving human participants was reviewed and approved by University of Graz. The participants provided their written informed consent to participate in this study.

## Author Contributions

IK-HW contributed to conceptualization, methodology, validation, investigation, formal analysis, resources, data curation, writing—original draft, writing—review and editing, visualization, and project administration.

## Conflict of Interest

The author declares that the research was conducted in the absence of any commercial or financial relationships that could be construed as a potential conflict of interest.

## Publisher’s Note

All claims expressed in this article are solely those of the authors and do not necessarily represent those of their affiliated organizations, or those of the publisher, the editors and the reviewers. Any product that may be evaluated in this article, or claim that may be made by its manufacturer, is not guaranteed or endorsed by the publisher.
